# A critical evaluation of methodological and mechanistic insights on probiotic-derived extracellular vesicles

**DOI:** 10.3389/fnut.2025.1632232

**Published:** 2025-08-29

**Authors:** Chantal Ronacher, Claudio F. Gonzalez, Graciela L. Lorca

**Affiliations:** Department of Microbiology and Cell Science, Genetics Institute, Institute of Food and Agricultural Sciences, University of Florida, Gainesville, FL, United States

**Keywords:** probiotics, bacterial extracellular vesicles, mechanisms, methodology, applications, study design, limitations, nutrition

## Abstract

Probiotic extracellular vesicles (pEVs) have emerged as promising postbiotics with potential applications in inflammatory diseases, infections, allergies, cancer treatment, autoimmune disorders, and even neurological and degenerative conditions. Yet despite the surge in research on pEVs, critical gaps and inconsistencies in study design, methodology, and mechanistic understanding hinder unlocking their full potential. This literature review provides a concise introduction to beneficial bacterial EVs, mechanistic insights into their role in interkingdom interactions, and current challenges in pEV research. We highlight methodological inconsistencies in model selection, control design, and effect measurement, discuss their consequences and provide recommendations to improve experimental rigor and comparability of results. These include methodological considerations like standardization strategies for pEV preparation, purification, formulation, and administration as well as general study design questions. Finally, we outline key avenues for future research, emphasizing the need for biomarkers to track pEV biodistribution, the identification of effector molecules, and a deeper understanding of their mechanistic targets, as well as their interactions with food components and their use as delivery systems, among others. By addressing these challenges, this review aims to provide a roadmap for advancing pEV research and facilitating their transition into clinical and biotechnological applications.

## 1 Introduction to probiotic-derived extracellular vesicles

Awareness about the importance of gut health in human well-being has increased in recent years, driven by dietary and wellness trends and a growing understanding of the microbiome’s role in maintaining health and preventing disease. Beneficial bacteria, commonly referred to as probiotics, have been recognized for their positive impact on host health, as well as their applications in agriculture and food preservation. Probiotics are defined as “live microorganisms that, when administered in adequate amounts, confer a health benefit on the host” and have been extensively studied for their roles in improving gut function, modulating immunity, and restoring microbial balance (1–8).

Most probiotic bacteria are Gram positive, with notable genera including *Bifidobacterium* and the *Lactobacillaceae* family, while Gram negative probiotics are primarily represented by *Escherichia coli* Nissle 1917 and *Akkermansia muciniphila* (9–11). Members of the *Lactobacillaceae* family belong to lactic acid bacteria group (LABs), producing lactic acid as a key fermentation product, which have long-standing use as starters in dairy products (12, 13). Despite the diversity of probiotic strains and their regular use, the mechanisms through which they exert their beneficial effects remain an active area of research.

Here we discuss the role of extracellular vesicles (EVs), nanoscale lipid particles secreted by live bacteria, in mediating these beneficial effects. EVs serve as carriers of bioactive molecules, including proteins, nucleic acids, lipids, and metabolites, enabling intercellular communication and interaction with the host (14–18). In 2019, the International Scientific Association for Probiotics and Prebiotics defined these structures as postbiotics (19). Collectively, vesicles of Gram negative bacteria are termed outer membrane vesicles (OMVs) while Gram positive vesicles are referred to as membrane vesicles or EVs in literature (20, 21). In this review, according to the guidelines provided by MISEV2018 (22), both are jointly called extracellular vesicles, except when addressing a specific subcategory. Probiotic-derived EVs (pEVs) have been implicated in enhancing intestinal barrier integrity, modulating immune responses, inhibiting pathogens, and influencing the host microbiome. Additionally, their ability to carry a diverse range of effector molecules to distant tissues suggests potential therapeutic applications beyond traditional beneficial effects (20, 21, 23–26).

Despite recent advancements, the study of pEVs is still in its infancy compared to that of pathogenic EVs, particularly of OMVs. The mechanisms underlying vesicle biogenesis, cargo selection, and host interaction remain incompletely understood. To date we recognize that vesiculogenesis, the process responsible for EV formation, involves genetic regulation. During this process, lipid bilayer structures bud from the cytoplasmic membrane and, in case of Gram positives, traverse the cell wall, a process during which cell wall modification is thought to play a crucial role. It is important to distinguish between EVs released in a controlled manner and structures formed after explosive cell lysis. Prophage-derived endolysins may aid in the release of EVs in both Gram positive and Gram negative bacteria (27) but also environmental conditions can stimulate vesiculogenesis as seen in the example of *Lactobacillus (L.) johnsonii* N6.2 where the presence of bile increased number of EVs by approximately 100-fold (28).

Research has further shown that EV composition differs from that of the parent bacterium, indicating specific functions and a potential sorting mechanism (15, 21, 29). Often a proteomic analysis of EVs either by electrophoresis in combination with LC–MS/MS or MALDI-TOF is included in studies. The results are relatively heterogeneous and usually report a magnitude of several hundred proteins associated with EVs (29). The majority of EV-related proteins were predicted to be cytoplasmic or plasma membrane proteins, with a smaller fraction predicted as cell wall or extracellular binding proteins. In pathogenic bacteria, these proteins often include toxins or other virulence factors transported within the vesicles. Conversely, for non-pathogenic strains, these proteins may contribute to mediating beneficial effects. For instance, *Lacticaseibacillus* (*Lc*.) *casei* BL23 has been found to carry proteins associated with its probiotic effects within its EVs (15, 21, 27, 29, 30).

As a representative of Gram negative probiotics, *Escherichia* (*E*.) *coli* Nissle 1917 has numerous adhesin and peptidoglycan rearrangement proteins that are enriched in its vesicle fraction. They are involved in the effective colonization of the host and its protection against pathogenic microorganisms. The protein content of EVs derived from *Lactobacillaceae* is dominated by metabolic proteins, which may indicate an effect in improving the digestive processes of the host (29). In general, EVs can carry a wide range of intraluminal cargo, including negatively charged phospholipids, nucleic acids, polysaccharides and proteins, influenced by the producer bacteria, growth and environmental conditions, as well as the mechanism of biogenesis (28, 31–33).

EVs in general can be taken up by recipient cells through multiple mechanisms, including clathrin-dependent endocytosis, caveolin-mediated uptake, macropinocytosis, phagocytosis, and lipid raft–mediated internalization. The specific uptake route may vary depending on the surface proteins and glycoproteins of both the EV and the target cell, and a single cell may use several pathways simultaneously to internalize a heterogeneous EV population (34). The uptake of bacterial EVs into eukaryotic host cells primarily occurs via endocytosis, whereby the exact pathway is influenced by the cell and vesicle type. Phagocytic cells use phagocytosis in addition to other mechanisms used by non-phagocytic cells such as epithelial cells. While EVs derived from Gram negative pathogens have been extensively studied for their uptake, limited information is available for Gram positive bacteria. Nonetheless, EVs from *Bifidobacterium longum*, *Clostridium butyricum*, and *Lactiplantibacillus* (*Lp*.) *plantarum* have been shown to be internalized by macrophages and dendritic cells via clathrin-mediated endocytosis and micropinocytosis, and *L. johnsonii* N6.2 EVs enter human beta cells via clathrin/dynamin-dependent endocytosis. Importantly, both studies use pharmacological inhibitors to dissect uptake pathways. While these methods provide valuable insights, they are subject to certain limitations. For example, pharmacological inhibitors may lack pathway specificity, potentially affecting multiple cellular processes, and fluorescent labeling can influence EV structure or function. In addition, findings from immortalized cell lines may not fully capture the complexity of *in vivo* systems (35, 36). Once internalized, EVs may escape the endosome to release effector molecules into the cytoplasm, initiating cellular signaling cascades and driving the expression of genes linked to immune regulation, antiviral defense mechanisms, or the detection of foreign RNA.

The exact mechanisms through which bacterial EVs overcome healthy epithelial barriers are not fully elucidated but may involve transcellular transport processes. Research indicates that EVs from gut-associated bacteria can reach various tissues, including the liver, muscle or brain. Their ability to traverse the mucus layer, travel to distant sites and interact with the host in a systemic way gives grounds to the importance of vesicles compared to their parent bacteria in clinical applications (37).

Consideration of methodological aspects, appropriate controls, experimental setting, and functional implications contribute to the robustness and applicability of research findings and facilitates the next step towards realizing their potential. The aim of this review is to compare and analyze existing work on the beneficial effects of bacterial EVs, focusing on their mechanism of action and methodology in research. We hope this will help with experimental design and interpretation of results in future work but also provide a concise introduction to the topic of the multifaceted beneficial functions of bacterial extracellular vesicles.

## 2 The specificity and mechanisms of EV interactions

**Figure 1:**
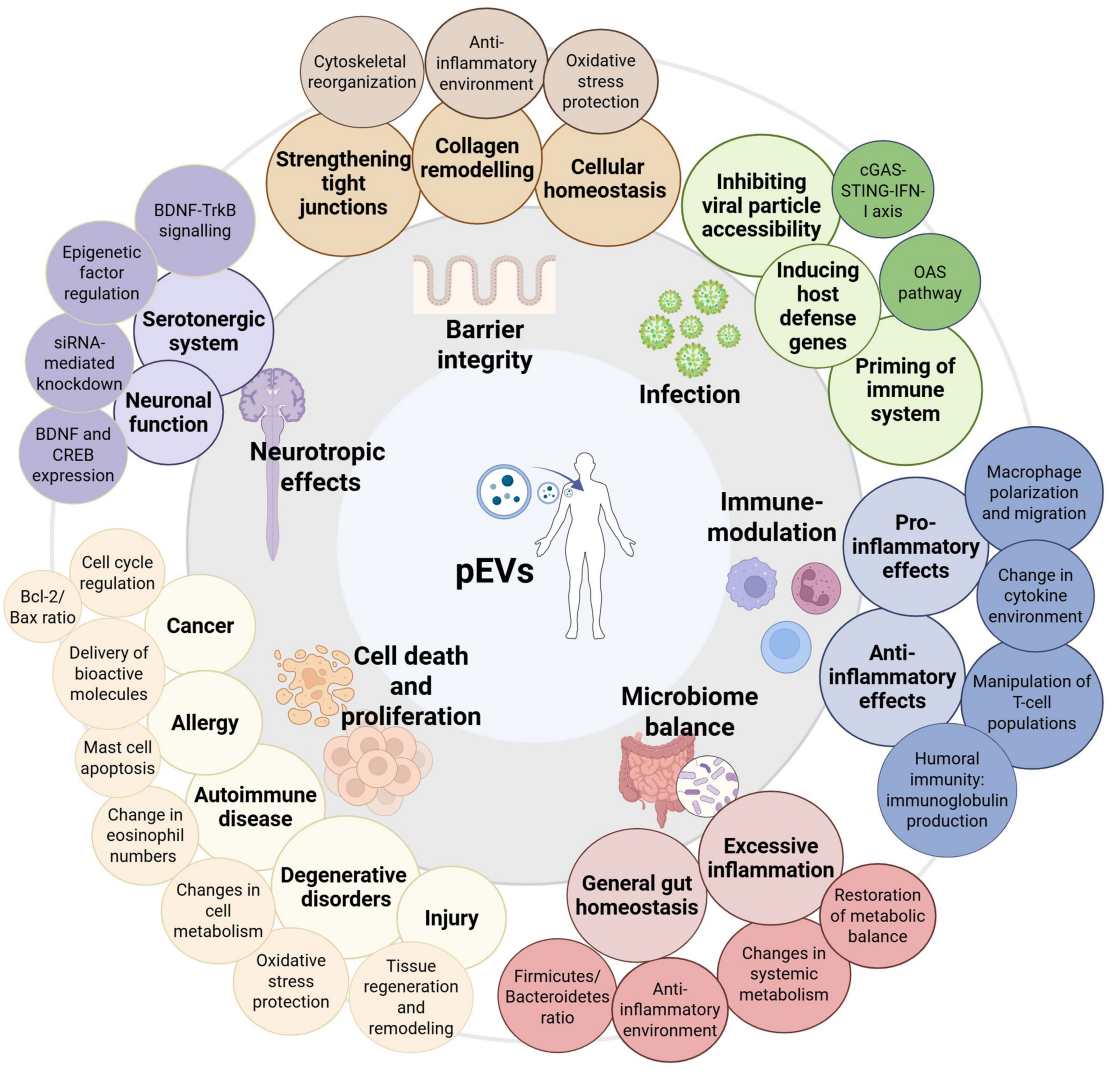
Graphic summary of the diverse biological effects of probiotic-derived EVs (pEVs) in health and disease including examples of identified mechanisms of action. The central image represents pEVs and their interactions with different physiological systems. The surrounding categories highlight key functional impacts, including barrier integrity protection (e.g., by strengthening tight junctions, collagen remodeling, and maintaining cellular homeostasis), protection against infection (e.g., inhibiting viral particle accessibility and activation of host defense), immune modulation (encompassing pro- and anti-inflammatory effects mediated by, e.g., cytokine environment changes), modulation of microbiome balance (e.g., maintaining gut homeostasis and restoration of metabolic balance), cell death and proliferation in diverse contexts (e.g., disease-related apoptosis, tissue regeneration or tumor regulation), and neurotropic effects (e.g., serotonergic system regulation, neuronal function modulation).

EVs from bacteria can mediate interspecies communication through several mechanisms. Naturally, EVs contribute to microbial survival by facilitating nutrient scavenging and microbial competition. In the context of direct host cell interactions, EVs can either be internalized or interact with surface-exposed components of host cells. The rupture of these vesicles can further lead to a local release of molecules influencing host responses. The ability of EVs to carry pathogen-associated molecular patterns (PAMPs) or microbial-associated molecular patterns (MAMPs), that are recognized by host pattern recognition receptors (PRRs) can enhance innate immune responses of the host by regulating the production of pro- or anti-inflammatory cytokines in immune cells (38, 39). This way these postbiotics may for example modulate the host immune response, enhance epithelial barrier integrity or reduce viral replication, highlighting their potential in controlling infections, maintaining gut homeostasis and preventing inflammation, as summarized in Figure 1 (37, 38, 40–42).

Much of the research on probiotics and their effector molecules has focused on their immunomodulatory properties, with both pro- and anti-inflammatory effects observed in several species. General priming of the immune system by introducing mild pro-inflammatory conditions is a frequently seen response achievable with both commensals (43) and probiotics (36, 44, 45). EVs are known to carry components such as lipopolysaccharides, lipoproteins, flagellin, and peptidoglycan, which can interact with host immune cells and activate downstream signaling pathways, including Toll-like receptor (TLR) and interferon signaling, among others (46). This priming can protect the host from infections (43, 47, 48) and can be mediated, for example, by enhancing macrophage killing activity and modulating T helper cell polarization (49), by delivery of bacterial DNA to distant host cells (via cGAS-STING-IFN-I axis) (50) or by modulating the gut microbiome (51–54).

Direct comparisons between the probiotic *E. coli* Nissle 1917 and the commensal ECOR12 revealed their potential to activate NOD1 signaling pathways in intestinal epithelial cells (IECs), demonstrating the role of both strains in controlled pro-inflammatory responses. These pathways involve the recognition of peptidoglycans released by OMVs, resulting in the activation of NFκB and the expression of inflammatory cytokines (43). On the other hand, *E. coli* Nissle 1917 could induce complex Th1/Th2/Th17/Th22/Treg responses via distinct dendritic cell activation while the secreted amounts of Treg- and Th1-triggering cytokines differed greatly for commensal *E. coli* vesicles (55). MicroRNA profiling of dendritic cells showed that intestinal *E. coli* activate the host’s innate immune system in a strain-specific manner. Vesicles from the probiotic *E. coli* Nissle 1917 induce Th1 responses critical for fighting pathogens and clearing infections, while the commensal ECOR12 OMVs program dendritic cells to orchestrate regulatory T-cells (Treg) responses to sustain immune tolerance in the gut (48).

The Gram positive *Lc*. *casei* EVs could also induce NFκB-mediated inflammation *in vitro* in unchallenged HT-29 human colon carcinoma cells. Two cell wall muramidases associated with EV-fractions of *Lc*. *casei* are p40 and p75. These proteins have been previously associated with the protective effect of the parent bacterium on the intestinal epithelium. The interactions of these vesicles with IECs lead to phosphorylation of the epidermal growth factor receptor on those cells and activation of the AKT pathway, ultimately resulting in NFκB-mediated inflammation (44). Interestingly, in unstimulated Caco-2 cells—another human colon carcinoma cell line—the EVs from *Lc*. *casei* BL23 increased production of anti-inflammatory cytokines, indicating specificity of mechanisms towards different cell lines (56).

In the context of disease, pEVs were consistently shown to dampen inflammation by contributing to a change in cytokine environment. Mostly these mechanisms are NFκB pathway dependent as well and lead to a re-establishment of an anti-inflammatory balance by reducing pro-inflammatory (incl. IL-6, IL-1b, IL-2 and TNF-*α*) and increasing anti-inflammatory (IL-10, TGFβ) signals (51, 53, 56–59). However, the suppression of inflammation can be achieved also indirectly through the polarization of macrophages and the manipulation of T helper cell populations (46, 55, 60–63). Many different pEVs of LABs have been observed to serve the overall same purpose, such as for example *Lc*. *rhamnosus* GG, *Lactobacillus kefirgranum* PRCC-1301, *Lp*. *plantarum* Q7, *Lc*. *paracasei* and kefir grain *Lactobacillus*, but also commensal bacteria, all relieving illness-related inflammation of the intestine (51, 53, 54, 57–59, 64). Simultaneously, the same EV can exert both pro- and anti-inflammatory properties, depending on the context. For example, *Lp*. *plantarum* has general immunostimulatory properties on healthy IECs while showing anti-inflammatory properties via macrophage polarization during skin inflammation (60, 65). In the context of colitis, an inflammation of the colon, *Lp*. *plantarum* Q7 EVs downregulated TLR4 and MyD88 expression, eventually leading to NFkB-mediated anti-inflammatory effects (51). The EVs of *Lc*. *rhamnosus* GG also modulate the immune system and the composition of the microbiota via the TLR4-MyD88 axis, altering the metabolism of the entire microbiome towards an anti-inflammatory environment, thus protecting against colitis in mice (53).

Just how diverse the functions of EVs can truly be is made even more clear by expanding the perspective beyond their immunomodulatory properties alone. For example, *Akkermansia* (*A*.) *muciniphila* OMVs have been used in the treatment of cancer, obesity, bone disease and depression (52, 61, 66, 67). Similarly, *Lp*. *plantarum* EVs have been applied in cancer therapy and stress-induced depression as well as during skin inflammation, ischemic brain injury and colitis (51, 60, 66, 68–71). Commensal *L. johnsonii* EVs were implicated in the mitigation of synovial inflammation and osteoarthritis, while *L. johnsonii* N6.2 EVs mitigate murine norovirus infection and symptoms of type 1 diabetes (62, 72, 73). *Lc*. *rhamnosus* GG EVs on the other hand have been investigated for the treatment of liver damage, intestinal inflammation and colon cancer (53, 64, 74, 75).

A closer look at the mechanisms by which these EVs perform such different functions reveals intricate interactions and potentially common pathways by which EVs can affect the host. Several EVs have been shown to induce a shift in microbiota leading to an anti-inflammatory and protective environment (51–54). For example, commensal bacteria and probiotic strains like *Lp*. *plantarum* and *Lc*. *rhamnosus* GG decreased the Firmicutes/Bacteroidetes ratio and restored gut homeostasis (64, 68, 75). In models of alcohol-induced liver injury, *Lc*. *rhamnosus* GG EVs restored gut taurine concentration indirectly via altering the gut microbiota, suppressing miR194 and activating the FXR-FGF15 pathway. Consequently, these actions led to the inhibition of bile acid *de novo* synthesis, lipogenesis, and prevented liver injury. Notably, administering EVs to normal-fed mice did not induce changes in measured parameters, highlighting the specific effectiveness of EVs under dysregulated conditions (64, 75). For an overview, pEVs of different origin together with their proposed functions and mechanisms are summarized in Table 1.

**Table 1:** Role of probiotic derived EVs (pEVs) in interkingdom interactions.

pEV origin organism	Function and/or mechanism observed	Disease/health context	Reference
Cytotoxicity			
*Bifidobacterium longum* KACC 91563	Apoptosis of mast cells (likely via family 5 extracellular solute-binding protein)	Food allergy	(81)
*Lacticaseibacillus (Lc.) paracasei* PC-H1	Upregulation of apoptosis genes (Bcl-2 /Bax)	Colorectal cancer	(80)
*Lc. rhamnosus* GG	Delivery of bacteriocins and polysaccharides	Hepatic cancer	(74)
*Lactiplantibacillus (Lp.) plantarum* CCARM 0067	Induce metabolic shift inhibiting cell proliferation (upregulation of p53 and p21)	Chemo resistant colorectal cancer	(70)
Anti-inflammatory			
*Akkermansia (A.) muciniphila* BAA-835	M1 macrophage recruitment	Prostate cancer	(61)
*Escherichia (E.) coli* Nissle 1917	Induce complex Th1/Th2/Th17/Th22/Treg responses via distinct dendritic cell activation	Health	(55)
*Lc. casei*	Increase in anti-inflammatory cytokines in unstimulated Caco-2 cells	Health	(56)
*Lactobacillus (L.) kefirgranum* PRCC-1301	NFκB pathway mediated change in inflammatory environment	Inflammation (colitis)	(57)
*Lp. plantarum* Q7	NFκB pathway mediated shift in inflammatory environment, down-regulated TLR4 and MyD88	Inflammation (colitis)	(51)
*L.* spp. (kefir grain)	Reduce pro-inflammatory cytokine release and counteract oxidative stress by decreasing myeloperoxidase serum levels	Intestinal inflammation	(59)
*Lp. plantarum* APsulloc 331,261	Stimulate a tolerogenic M2b macrophage type	Skin inflammation	(60)
*L. johnsonii* sp.	Stimulate a tolerogenic M2b macrophage type	Synovial inflammation	(62)
*L. johnsonii* sp.	Stimulate macrophage glutamine synthetase/mTORC1 pathway	Degenerative disease/osteoarthritis	(62)
*L. johnsonii* N6.2	Induce AHR translocation and STAT3 activation; increase in insulin production and glucose uptake in pancreatic islets only under high glucose condition; induce a tolerogenic M2b phenotype	Autoimmune disease/Type I diabetes (T1D)	(73)
*Pediococcus pentosaceus*	TLR2 mediated secretion of IL-10, secondary effects on macrophages and T cells	Injury/wound healing	(63)
*E. coli* 083	TLR2/4/5 and NOD1/2 interactions; decrease eosinophil numbers; reduce Th2 cytokine production and mucus secretion; decrease in allergen-specific IgA and IgE levels	Airway allergy	(46)
*Lc. rhamnosus* GG	Shift in metabolism of the microbiome (increase valine, leucine and isoleucine degradation and decrease fluorobenzoate degradation) via TLR4-MyD88 axis	Intestinal inflammation	(53)
Immune system priming			
*E. coli* ECOR12	Activate NOD1, activation of NFκB pathway via peptidoglycans released by OMVs	Health	(43)
*E. coli* ECOR12	Stimulate Treg responses to sustain immune tolerance	Health	(48)
*E. coli* Nissle 1917	Activation of macrophages modulating Th cell polarizing cytokines and oxidative stress via inducible nitric oxide synthase (iNOS)	Health	(49)
*Lc. casei* BL23	EV-derived p40 and p75 stimulate phosphorylation of EGFR, AKT pathway activation; NFκB mediated inflammation	Health	(44)
*Latilactobacillus sakei* NBRC 15893	TLR2 mediated increase of nitric oxide and retinoic acid	Health	(101)
*E. coli* O83	NFκB pathway activation via TLR4, TLR2 and TLR5 activation via flagellin receptor	Health and allergy	(46)
*Lp. plantarum* JCM8341	NFκB pathway activation by TLR1/2 or TLR2/6 heterodimers; EV-derived Lp19180 identified as effector molecule for TLR2 recognition; activate innate and acquired immune responses	Health	(65)
Pro-inflammatory			
*Limosilactobacillus* (*Lim*.) *reuteri* DSM 17938 and BG-R46	NFκB pathway mediated change in inflammatory environment including immune priming and anti-inflammatory effects	Bacterial infection	(47)
*A. muciniphila* BAA-835	Activate macrophages likely through delivery of PAMPs and antigens to TLR and NOD-like receptors	Prostate cancer	(61)
Intestinal barrier protection			
*A. muciniphila* MucT (BAA-835)	Decrease Angptl-4 in colon, adipocyte size and decrease lipid homeostasis; reduce alanine aminotransferase and aspartate aminotransferase (AST) in plasma, decrease expression of fatty liver related genes	Inflammation (obesity)	(52)
*Lc. rhamnosus* GG	Increase TJ proteins and Nrf2 and Nqo1; decrease ROS, inhibit NFĸB and improve antioxidant defenses	Inflammation (alcohol-induced liver injury)	(75)
*E. coli* Nissle 1917	Improve barrier integrity, dcrease *MMP-9;* induce recovery of TFF-3, TcpC- independent; upregulate claudin-14 and ZO-1; downregulate claudin-2	Inflammation (colitis)	(94)
*L. kefirgranum* PRCC-1301	Improve barrier integrity by inducing ZO-1, claudin-1, and occluding	Inflammation (colitis)	(57)
Microbiota composition			
Commensal bacteria (*Clostridium butyricum* MIYAIRI 588)	Decrease the Firmicutes/Bacteroidetes ratio	Intestinal inflammation and obesity	(54)
*Lc. rhamnosus* GG	Restore gut taurine concentration via altering the gut microbiota; suppress miR194 and activate FXR-FGF15 pathway; inhibit bile acid *de novo* synthesis and lipogenesis	Inflammation (alcohol-induced liver injury)	(64, 75)
*Lc. plantarum* KCTC 11401BP	Decrease the Firmicutes/Bacteroidetes ratio	Inflammation (dermatitis)	(68)
Neurotropic effects			
*Lp. plantarum* KCTC 11401BP	Increased expression of Bdnf and Sirt1 via siRNA-mediated knockdown; stimulate BDNF and CREB expression	Depressive behavior (neuronal function)	(71)
*A. muciniphila* BA-835*, Bacillus subtilis* var. natto*, Lp. plantarum* KCTC 11401BP	Restore epigenetic factors regulating Bdnf and Nt4/5 expression in hippocampal cells by pathways involving MeCP2, HDAC2, and Sirt1; BDNF–TrkB signaling in the limbic system via the tryptophan-kynurenine pathway	Depressive behavior (serotonergic system)	(66)
*A. muciniphila* BAA-835	EV interactions in the gut modulate the host serotonin or 5-hydroxytryptamine system	Health (serotonergic system)	(76)
Oxidative stress and cell death protection			
*Lc. paracasei* sp.	Suppress iNOS activation leading to decreased COX-2, iNOS and NFκB and NO	Intestinal inflammation	(58)
*Lp. plantarum* YW11	Reduce apoptosis (Bax/Bcl-2, caspase3) in ischemic neurons via microRNA-101a-3p/c-Fos/TGF-*β* axis	Ischemic brain injury	(69)
*L. johnsonii* N6.2	Decrease in oxidative stress markers in primary human pancreatic islets, increase GLUT6 and SREBF1 mRNA levels	Autoimmune disease (T1D)	(73)
*L. crispatus* BCRC14618	Restore Akt phosphorylation to reverse cell death caused by oxidative stress via elevated Mfn-2 expression in 3A-sub-E placental cells	Spontaneous preterm birth	(79)
Protection from infections			
*L. gasseri* BC12 and *L. crispatus* BC3	Inhibit HIV-1 viral particle accessibility	Viral infection	(84)
*L. johnsonii* N6.2	Reduce murine norovirus replication in macrophage and mice model; activate the 2′,5′-oligoadenylate synthetase pathway; SH3b2 domain of Sdp in EVs identified as the effector protein	Viral infection	(72)
Commensal bacteria (*E. coli* MC1061*, Bacteroides fragilis* NCTC9343 and *Enterococcus faecalis* OG1RF)	Systemic protection via cGAS-STING-IFN-I axis (delivery of bacterial DNA)	Viral infection	(50)
Commensal bacteria (*Enterobacter cloacae* ATCC 13047, *Bacteroides thetaiotaomicron* ATCC 29148, *Salmonella typhimurium* UK-1 ATCC 68169)	Systemic protection via activating general antiviral immune responses	Viral infection	(82)
*Lp. plantarum* WCFS1	Increase expression of host defense genes (cpr-1 and clec-60 and human homologs)	Bacterial infection	(83)
Tissue reorganization and cell proliferation			
*A. muciniphila* BAA-835	Alter bone metabolism and prevent bone loss	Bone disease	(67)
*Ligilactobacillus animalis* ATCC 35046 (and *Lim. reuteri* ATCC PTA 6475)	Inhibit apoptosis and stimulate osteogenesis	Bone disease	(77)
*L. druckerii* sp.	Decrease collagen I/III and *α*-SMA and lower cell proliferation of fibroblasts; increased proliferation of skin cells and new blood vessel formation	Injury/wound healing	(78)

EVs from the three different probiotics *Lp*. *plantarum*, *A. muciniphila* and *Bacillus* (*B*.) *subtilis* could produce anti-depressive-like effects in Chronic Restraint Stress Treatment (CRST) mice by using slightly different but overlapping pathways, all restoring epigenetic factors regulating brain-derived neurotrophic factor Bdnf and Nt4/5 expression in hippocampal cells. Authors speculate BDNF–TrkB signaling in the limbic system via the tryptophan-kynurenine pathway as mechanism of action (66). The impact of *A. muciniphila* EVs on the serotonergic system was separately tested *in vivo* in mice. After oral administration of vesicles serotonin levels increased in hippocampus and colon tissues but decreased in serum, accompanied by a change in the inflammatory cytokine profile of the colon. These results suggest that *A. muciniphila* EVs can influence serotonergic pathways, potentially affecting mood and behavior (76). Together this evidence highlights how probiotic EVs uniquely affect brain function and behavior through specific molecular mechanisms, showcasing their potential to influence the gut-brain axis.

Probiotic EVs often seem to have common approaches of reaching the same outcome, independently of parent bacterium species and specific disease context. The reorganization of tissue for example to aid in wound healing or alleviate bone disease reports three distinct probiotics—*A. muciniphila*, *Ligilactobacillus animalis* and *Lactobacillus druckerii*—all stimulating cell proliferation to build up and reorganize tissue like the formation of new blood vessels (67, 77, 78). The prevention of cell death by protection against oxidative stress in different cell types such as placental cells, neurons or in insulin-producing cells is another example of how different EVs utilize similar mechanisms in varying disease contexts (69, 73, 79). *L. crispatus* EVs were investigated in the treatment of spontaneous preterm birth. Both, cell senescence and death in placental cells, caused by oxidative stress induction, could be reversed via recovery of AKT phosphorylation in the cells as tested *in vitro*. Mitofusin-2, a mitochondrial membrane protein regulating mitochondrial fusion, was overexpressed in cells receiving H_2_O_2_ and EV-treatment. Therefore, EVs lead to an attenuation of mitochondrial fission in H_2_O_2_-induced placental cells (79). *Lp*. *plantarum*-derived EVs could protect against ischemic brain injury after crossing the gut-brain axis. Bacterial EVs reduced apoptosis (Bax/Bcl-2, caspase3) in ischemic neurons via the microRNA-101a-3p/c-Fos/TGF-*β* axis both *in vivo* and *in vitro* (69).

In an *in vitro* model of type 1 diabetes, *L. johnsonii* N6.2 derived EVs could protect pancreatic beta cells from apoptosis in a dose-dependent manner by activating the non-canonical Aryl Hydrocarbon Receptor (AHR) and 2′,5′-oligoadenylate synthetase (OAS) pathways, leading to oxidative stress protection and inducing anti-inflammatory pathways (73). Interestingly, the pathways triggered seem to be context dependent. *L. johnsonii* N6.2 EV administration to macrophages induced a tolerogenic M2b phenotype mediated by activation of STAT3. A co-culture experiment further revealed an intricate interplay where stimulation of human monocytes with EVs increased the IL-10 expression in pancreatic beta cells via STAT3 activation (73).

The modulation of macrophage populations towards a M2b tolerogenic phenotype—as with *L. johnsonii* N6.2 EVs—is a common mechanism by which EVs can soothe severe inflammation of the host. In an acute inflammation model, *Pediococcus pentosaceus* EVs reduced inflammation by polarizing bone marrow derived macrophages towards a tolerogenic M2b phenotype and by promoting myeloid-derived suppressor cell differentiation in bone marrow progenitors, likely in a TLR2 dependent way. Resulting cells secreted less TNF-*α* and IL-6 compared to lipopolysaccharide (LPS) stimulated cells (63). Similarly, during skin inflammation *Lp*. *plantarum* EVs could stimulate a tolerogenic M2b type (60) as in synovial inflammation (62).

The fact that EVs can exert the opposite effect, depending on the context, illustrates their specificity for the respective disease state. For example, *A. muciniphila* was shown to have tumor killing properties by recruiting an increased proportion of M1 macrophages to the tumor site, likely by delivery of PAMPs and antigens to TLR and NOD-like receptors of immune cells (61). While EVs can provide protection against cell death in the context of immune diseases or injury, several EVs have shown cytotoxic effects in the context of cancer or allergy. They can regulate the expression of apoptosis gene family Bcl-2 and Bax, deliver bacteriocins and polysaccharides or, in chemo-resistant colorectal cancer cells, reverse the metabolic shift to restore chemosensitivity via the PDK2-mediated glucose metabolic pathway, eventually leading to apoptosis (70, 74, 80).

Further, the probiotic *Bifidobacterium longum* KACC 91563 derived EVs have shown very promising and specific cytotoxic effects only on mast cells during food allergy. The family 5 extracellular solute-binding protein, proposed to be found on the surface of these EVs, was speculated to be a driving effector molecule in this interaction (81). In airway allergies, *E. coli* 083 OMVs could lower eosinophil numbers in the lung and reduce allergen-specific IgA and IgE levels in mice models. The vesicles were shown to interact with PRRs TLR2/4/5 and NOD1 and NOD2, reducing Th2 cytokine production and mucus secretion, ultimately reducing allergy symptoms (46).

Based on the research reported so far, it has become increasingly evident that some mechanisms occur repeatedly and show commonalities between species and function, albeit achieved through slightly different pathways. A common mechanism of pEVs that results in host protection is through the stimulation of responses that lead to the inhibition of pathogen’s insults. This can be observed in immunomodulatory actions such as priming macrophages and T-helper cells but also by inducing the expression of host defense genes (47, 49, 50, 82, 83). Noteworthy examples where EVs can protect the host from infections include commensal bacterial EVs effectively controlling murine norovirus infection by modulating antiviral immune responses (82) and symbiotic vaginal *Lactobacilli* EVs. It has been reported that EVs from *L. crispatus* BC3 and *L. gasseri* BC12 inhibit HIV-1 infection by impeding viral particle accessibility (84). Moreover, *L. johnsonii* N6.2-derived EVs were shown to activate the OAS pathway, inducing the expression of antiviral proteins, resulting in a significant reduction of murine norovirus replication in macrophage cells and in the murine model of infection. The SH3b2 domain of Sdp, a protein specifically enriched in *L. johnsonii* N6.2 vesicle fractions, has been identified as the sole effector protein driving this pathway activation, demonstrating that proteins enriched in EVs can precisely orchestrate host responses to control viral infections (72). Understanding the effector molecules present in EVs and their interactions with host cells is crucial for uncovering their mechanisms of action. Unfortunately though there are currently few reports focusing on the identification and characterization of these factors.

Overall, the intricate balance between opposing responses like pro- and anti-inflammatory properties emphasizes the need for a nuanced understanding of the mechanisms involved in the interactions between bacterial EVs and the host immune system. The context-dependent specificity of beneficial EV-mediated effects even beyond the immune system warrants critical evaluation as well. Examples include *Lp*. *plantarum* EVs reducing apoptosis in neurons but inducing apoptosis in 5-fluorouracil-resistant cancer cells (69, 70) as well as *A. muciniphila* EVs decreasing cell death in diseased bone while suppressing the proliferation of cancerous cells (61, 67). In contrast, comparable effects of different probiotic strains have also been reported, e.g., in wound healing models or in cancer progression on different cell lines, which supports the comparability of the mechanisms by which beneficial EVs act on host cells (61, 63, 70, 74, 78, 80, 85). Similarly, complications such as fibrosis arising from various diseases in altered tissues have been reported. Nevertheless, EV treatments of different bacteria of origin resulted in comparable effects, indicating an outcome specific to a particular condition in the host (63, 78, 85, 86). Still, comparing these findings remains challenging due to the absence of mechanistic targets and a lack of understanding of the bioactive components. While many scientific reports suggest potential pathways there is an urgent need for further mechanistic research in this area.

## 3 Methodologies used in EV isolation and administration, and the importance of EV distribution

### 3.1 Preparation and formulation of EVs

The therapeutic potential of bacterial EVs depends on their isolation, administration, and distribution within host systems. However, at present, inconsistencies in methodologies pose challenges for standardizing outcomes and interpreting results across studies.

Isolation of bacterial EVs typically involves sequential steps of bacterial cell cultivation and removal of intact cells followed by vesicle concentration and purification. Common techniques include ultracentrifugation, ultrafiltration, and size-exclusion chromatography but these methods continue to be marked by a lack of standardization, leading to variations in the size and subpopulations of isolated EVs (87, 88). While influenced by bacterial strain and analytical technique, Gram negative-derived EVs typically exhibit dimensions in the range of 20 to 200 nm, while Gram positive-derived EVs show a broader range of 50 to 300 nm diameter (29). Additional sources of variability include exosome contamination from components of the culture media, differences in media composition, and the bacterial growth phase at which EVs are harvested (87, 88). These inconsistencies may impact their function and biodistribution, making it difficult to compare results across studies. Addressing these challenges requires consistent protocols that ensure reproducibility and enable meaningful comparisons, alongside transparent and accurate reporting of methods.

Techniques for the quantification of EVs also vary, with most studies using relative protein abundance, while others count particle numbers (62, 89, 90). Standardizing EVs by protein concentration can be misleading due to variations in cargo and surface protein load caused by different growth conditions. Normalizing by particle number may reduce this variability, ensuring more consistent comparisons between samples, while standardization of EVs per colony forming units directly links vesicle production to bacterial cell number, providing a complementary perspective on EV yield relative to bacterial population. However, relating EV dose to bacterial cell number remains challenging because few studies establish a direct equivalence between EVs and live bacteria. For example, in a murine norovirus infection model, a dose of approximately 1 × 10^10^ EVs yielded similar beneficial effects as 1 × 10^8^ CFUs of *Lactobacillus johnsonii* N6.2 (72). Noteworthy, while EVs can mimic some of the beneficial effects of live bacteria, their transient nature would require repeated administration, whereas live bacteria may colonize and continuously release EVs.

But this is not the only source of deviations in final applied EV-concentration. For example, in *in vivo* cancer-related experiments, two separate studies, one on prostate cancer (*A. muciniphila*) and one on colorectal cancer (*Lc*. *paracasei*) administered each 40 μg of EVs per mouse. However, *A. muciniphila* EVs were injected intravenously after tumor formation while *Lc*. *paracasei* EVs were injected subcutaneously together with the tumor-inducing HCT116 colorectal cancer cells. Both experiments showed a decrease in tumor burden (61, 80). *In vitro* studies on hepatic and chemo-resistant colorectal cancer cells, in contrast, demonstrated substantial differences in administered concentration, with 50–200 μg/mL *Lc*. *rhamnosus* GG EVs or 0.1–10 μg/mL *Lp*. *plantarum* EVs, respectively (70, 74). Importantly, *Lc*. *rhamnosus* GG EVs only showed a cytotoxic effect with 100 μg/mL for 24 h, while other concentrations did not yield the same results, underlining the importance of including a relatively broad range of concentrations especially if there is no prior work to base estimates on.

Another example demonstrating that the effective EV formulation may be different between species is a study by Choi et al., where standardized protein concentrations were used to assess the impact of different pEVs’ anti-depressive effects (66). Here, the fold change in expression of neurotropic factor-regulators *in vitro* in hippocampal cells was more pronounced with *B. subtilis* EVs compared to those from *Lp*. *plantarum* or *A. muciniphila*. Contrastingly, *in vivo*, the impact of *B. subtilis* EVs on the same genes was similar to other bacterial families tested, pointing out the different effective concentrations *in vivo* and *in vitro* (66). Another study explicitly studying the effects of *Lp*. *plantarum* EVs in the same context was very similar in outcomes, however the administered concentration of EVs was approximately 0.0020–0.0025 μg (0.1 μg/kg) as compared to 6 μg of protein (approximately 240–300 μg/kg) in the study by Choi et al.; highlighting a magnitude difference of ca. 10^3^ times (66, 71). Despite the significant difference in dosages, both studies reported antidepressant-like effects following EV treatment, though the specific mechanisms and potential dose-dependent effects would require further investigation to be fully understood.

The impact and range of concentration effects can also be seen in the context of intestinal inflammation, where the use of *L. kefirgranum* EVs on DSS-induced colitis led to more pronounced effects on intestinal integrity at higher (3.0 mg/kg) doses as compared to lower doses (0.03 mg/kg), while for example *Lp*. *plantarum* Q7 EVs did not show significant differences in outcome between 0.5 and 1.0 mg/kg (51, 57). *In vitro* experiments on intestinal epithelial cells also showed dose-dependent effects, for example on cytokine profiles, toll-like receptors, and tight junctions in concentration ranges as small as 0.1 to 5.0 μg *A*. *municiphila* EVs (91) and between 100 and 150 μg/mL *Lc*. *casei* EVs (56).

Overall, while the rationale behind the dosage form is often not clearly stated in the reports, the evaluation of those has a significant impact on the results, as dose-dependent effects are often seen and contribute to the rigor and confidence on the results observed.

### 3.2 Timing of EV-administration and delivery mode

Experiments evaluating the positive impact of bacterial EVs on wound healing investigated wound treatment via subcutaneous injections, direct topical application, or intraperitoneal injections, post-wounding, across studies. Results consistently demonstrated a beneficial effect of EVs on wound healing, whether the treatment occurred only twice over 12 days, six times over 15 days, or four times over 7 days when administered through injections. Notably, topical treatment every other day did not lead to improved healing compared to untreated controls (63, 78, 85). Despite researchers universally reporting accelerated healing via systemic administration, the mechanisms described exhibited slight variations, suggesting the presence of strain-specific effects that partially overlap. Taken together, these examples highlight that the overall functions of a diverse group of bacterial EVs are very similar in a narrow context and that different bacterial sources can lead to the same overall results despite different mechanisms, whereas the route of administration had a clear impact on effectiveness of the treatment (63, 78, 85). It is worth to mention the importance of simplicity in application in terms of standard clinical practice, which would make direct application options an interesting area to explore further. This was addressed very recently by Kuhn et al. (32), who embedded *Lp*. *plantarum* and *Lc*. *casei* EVs in a hydrogel matrix for cutaneous application to accelerate wound healing.

The importance of timing of EV-treatment is showcased in a study testing the effects of *Lp*. *plantarum*-derived EVs in a model of dermatitis, an inflammation of the skin (68). *In vitro* tests included assessing the immunogenicity of EVs on untreated cells and their protective effect when pre-treated—before stimulation with *S. aureus* induced EVs—versus co-treated. Results demonstrated a dose-dependent preventive effect of *Lp*. *plantarum* EVs on skin inflammation while co-treatment groups showed no significant impact. In 2022, Chen and team (77) compared preventative (co-treatment) with therapeutic (post-treatment) treatment options in osteonecrosis, revealing that probiotic EVs could only alleviate symptoms in early stages of the disease. Unlike many other studies focusing on a single mode of administration, this article underscores the substantial influence of administration timing on outcomes. The choice of treatment option must be well-reasoned and clinically relevant. Especially with bone diseases like osteoporosis and osteoarthritis increasing in risk with age, a preventative treatment is a realistic and promising option (92, 93). The study also included controls for EV-treated healthy cells and mouse models, demonstrating that some effects are consistent in both healthy and diseased conditions, while others are specific to either. This holds particular significance in the context of preventative treatments, where the condition may not have fully manifested at the point of administration, potentially leading to unintended side effects if controls are not established during development.

The timing of effect-measurement is equally critical, as effects may be noticeable shortly after the treatment but not several days post-intervention as demonstrated by the change in fibrosis markers and an attenuation of hypertonic scar formation by administration of *L*. *druckerii* EVs on a scleroderma mouse model. Changes in mRNA and protein levels of fibrosis markers were evident 24 to 48 h post-treatment. In contrast, a study applying *Synechococcus elongatus* PCC 7942 EVs on wound healing and associated differentiation of dermal fibroblasts did not assess early changes, but after 12 days, no significant differences in those markers were detected (63, 78, 85).

Lastly, the location of measurement may impact the interpretation of results and should be mechanistically reasoned. For example, oral administration of *A. muciniphila*-derived EVs increased serotonin levels in the hippocampus and colon of mice, but decreased them in the serum, reflecting the importance of monitoring tissue-specific as well as systemic markers to capture localized effects in target tissues (76). In general, understanding the biodistribution of EVs is critical for assessing their therapeutic mechanisms and off-target effects. Studies on bone diseases have employed oral, rectal, or intravenous routes, demonstrating systemic vesicle distribution to the liver, spleen, brain, muscle, lungs, gastrointestinal tract, kidneys, and bones within an hour of administration (62, 67, 77). However, these studies relied on labor-intensive methods such as fluorescent labeling or polyclonal antibody production to trace EVs, highlighting the urgent need for reliable biomarkers.

In summary, the studies reviewed emphasize the critical need for standardized and well-reasoned administration protocols. This is particularly crucial given the variations in concentrations and measurement methodologies. Moreover, the significance of treatment timing has been clearly demonstrated, as seen in the comparison between preventive and post-treatment options for bone diseases. Additionally, the location of measurement plays a pivotal role, with distinct results obtained when measuring cytokine levels in serum compared to tissue samples. Although the information about EV distribution obtained using fluorescent labels from the experiments mentioned above is exceptionally valuable, existing studies lack well-established biomarkers for observing the systemic distribution of specific vesicles, with only one publication directly addressing this concern to the best of our knowledge, which has identified Sdp as biomarkers for the systemic distribution of EVs derived from *L. johnsonii* N6.2 (33).

## 4 Studying the role of EVs in interkingdom interactions

### 4.1 The choice of model, controls and measurement method

Research into bacterial EVs has utilized both *in vivo* and *in vitro* models to assess their impact on immune responses and intestinal health. *In vivo* studies, often using murine models of gastrointestinal disease, such as colitis in mice, have demonstrated that EVs from probiotics and commensal bacteria can alleviate symptoms, restore gut homeostasis, and regulate inflammation (51, 57, 58, 63, 94). For example, *L. johnsonii*-derived vesicles have been shown to inhibit macrophage migration and induce a tolerogenic immune profile by manipulating the M2/M1 macrophage ratio (62, 73). On the other hand, EVs from *E. coli* Nissle 1917 promote stronger bacterial killing activities in macrophages and modulate downstream cytokine production, highlighting their role in enhancing host defense mechanisms (49). Furthermore, in models of inflammatory bowel disease (IBD), EVs derived from gut commensals have demonstrated significant protective effects by regulating inflammation, restoring immune balance, and enhancing intestinal barrier integrity (51, 57, 58, 63, 94). Mouse models are historically useful for studying the immune system in health and disease because of their genetic similarity to humans, their ease of manipulation, and their well-characterized immune responses (95). However, pEV-studies are even extended to other animals, such as chickens, reinforcing the broad applicability of EVs in diverse biological systems (96).

In contrast, *in vitro* experiments offer controlled environments to study EVs’ effects on specific cell lines. Studies use different intestinal epithelial cell lines or monolayers to investigate the direct effects of EVs on inflammation. HT-29 and Caco-2 are both human colorectal adenocarcinoma cell lines commonly used to model the intestinal epithelium *in vitro*. While Caco-2 cells produce no mucin but often stronger tight junctions, HT-29 cells can build a mucus layer that influences pathogen adhesion and invasion (97). To trigger inflammation in the model, proinflammatory inducers such as LPS, tumor necrosis factor-alpha (TNF-*α*), dextran sodium sulfate (DSS), or IL-1β are applied (57–59, 89). One study, specifically comparing the effects of EVs on inflammation in HT-29 cells, revealed a specificity to stimulation with LPS compared to IL-1β or TNF-α, prompting a critical examination of studies that assume a single inducer is adequately representative of inflammatory conditions (89).

Several studies have explored the protective impact of EVs on intestinal barrier integrity, particularly in IBD. Both *in vitro* and *in vivo* gene expression analysis and protein level assessments consistently show that probiotic EVs enhance the integrity of tight junctions (TJs) crucial for maintaining intestinal barrier function (37, 40). For example, *E. coli* Nissle 1917 OMVs, when tested in DSS-induced colitis models, observed protein levels of TJ proteins like claudin-14 and ZO-1, which correlated well with expression levels, indicating a transcriptional regulation of TJ proteins (94, 98, 99). Beyond IBD, barrier protective functions of EVs have been studied in various conditions such as obesity (52), alcohol-induced liver injury (75), *Staphylococcus aureus* challenged IECs (47), and infections with enteropathogenic *E. coli* (EPEC) (100), consistently showing alleviation of symptoms. Notably, during EPEC infection, barrier dysfunction was reversed through the reorganization of proteins associated with TJ function (such as ZO-1 and occludin) and the cytoskeleton (e.g., F-actin), without corresponding changes in mRNA expression profiles. This finding highlights the importance of comprehensive assessments of EV-induced effects on different levels of expression and organization.

In general, the measurement of parameters such as cytokine levels during inflammation, should be standardized or expanded to include mRNA as well as protein levels in culture supernatants, tissues, or serum. Cytokine levels across studies were mainly measured with RT-qPCR or ELISA in cell culture supernatants (56, 57, 62, 89, 90) or in colon tissues and serum (51) while intestinal barrier markers were measured either on mRNA or protein level (57, 91) in cell cultures or tissue sections. Most information can be drawn from experiments combining several of these methods, as has been exemplified earlier. This comprehensive approach will contribute to a more nuanced understanding and facilitate the translation of promising findings into effective clinical applications.

A notable gap in current research lies in the limited direct comparison between EV treatments on healthy and diseased tissues or cells, highlighting the need for future investigations to aid in contextualizing these outcomes. Research on healthy intestinal epithelial cells has shown both anti-inflammatory and pro-inflammatory effects depending on the EV dose and bacterial strain (43–45, 48, 56, 91, 101). Some studies partially replicated the effects observed during inflammation, such as the augmentation of TJ proteins or the creation of a tolerogenic anti-inflammatory environment, while others revealed opposing effects, often in a dose-dependent manner, as summarized in Table 1 (102–104). Several studies have undertaken comparative analyzes between commensal bacteria and probiotic strains, showcasing strain-specific or general responses (43, 48, 55, 103). For instance, comparing commensal *E. coli* strains with probiotic *E. coli* Nissle 1917 revealed that the probiotic strain induced a more intricate T cell response, while the commensal strains seemed to suppress Th1 responses to LPS (48). A frequently observed response is the general priming of the immune system, a phenomenon achievable with both commensals and probiotics (43–45).

Also, the comparison of effects between parent bacterium and treatment with purified EVs can give valuable information on the mechanistic effects, such as for example *Lc*. *casei* was shown to increase TLR9 gene expression as well as IL-10 and IFNγ levels in Caco-2 cells whereas EVs significantly decreased TLR9 gene expression and decreased the level of IFNγ, while raising the level of anti-inflammatory cytokines (56). More rarely seen is an explicit comparison of EV-treatment to stimulation with cell-free supernatant, cell-EV-free supernatant, or with the media and purified protein, to evaluate for putative active factors secreted in a soluble form and their specific active components (99). A good example is *Lc*. *rhamnosus* JB-1 EVs, which are enriched for heat shock protein components like chaperonins, where administration of a purified protein could reproduce regulatory and neuronal effects *in vitro* and *in vivo* (102). In a model of norovirus infection, similar efficacy was observed between *L. johnsonii* N6.2 whole cells, derived EVs and SH3b2 proteins in liposomes, both in macrophage cell lines and a mouse model (72). Finding these specific bioactive molecules associated with EV-function is an essential step towards their safe and efficient therapeutic application.

### 4.2 Understanding systemic effects, synergy and the need for biomarkers

Naturally occurring EVs from several bacteria were investigated for their potential in treatment of cancer (61, 70, 74, 80). In the context of hepatic and colorectal cancer an increase in apoptosis markers after incubation with EVs was observed, indicating a cytotoxic effect of *Lactobacillus*-EVs on cancer cells. *Lp*. *plantarum* EVs were specifically tested for their anti-proliferative effect on chemo-resistant colorectal cancer cells, revealing no such impact on non-resistant HCT116 cancer cells. *In vivo* experiments including *A. muciniphila* and *Lc*. *paracasei* EVs also integrated controls for cytotoxicity on healthy cell lines and tissues, revealing no significant damage to non-tumor tissues. While both studies administered EVs systemically, the timing and duration of EV-administration varied. In the prostate cancer model, EV-administration commenced after tumor development and persisted for 13 days, while in the colorectal cancer model, EVs were co-administered with tumor-inducing HCT116 cells only once (61, 70, 74, 80).

Despite variations in probiotics, administration methods, and mechanisms these experiments demonstrate comparable end-results. However, recognizing the diversity of functions of the same probiotic EVs in different contexts underscores the importance of incorporating controls for systemic effects. Addressing this need, biomarkers to track EV-distribution in the body would be highly beneficial. For instance, a study by Bulut et al. (63) showcased worsened tumor formation due to commensal EVs suppressing cell-mediated immunity against the vaccinated tumor antigen, further emphasizing the importance of understanding off-target effects. Especially in the treatment of cancer, co-administration with other tumor-growth inhibiting medications may be relevant, and the impact of EVs on drug-induced weakened immune systems warrants consideration, given their earlier demonstrated influence on immune responses. In contrast, co-treatments with EVs of different origins could potentially activate complementary pathways, leading to increased therapeutic effects. One very recent study investigated the combination of *Lc*. *rhamnosus* GG EVs with anti-PD-1 immunotherapy against colorectal cancer and reported an increase in the therapies’ efficiency (105). This promise of exploring combination therapies has moreover been shown by a study comparing three kefir-derived *Lactobacillus* EVs—*L*. *kefir*, *L. kefiranofaciens*, and *L. kefirgranum*—that found a synergistic effect improving IBD when used in combination (59). This suggests the potential merit of investigating synergies between different strains or bacterial sources in future therapeutic strategies. Lastly, we want to mention the emergence of engineered EVs as, for example, cancer drug delivery systems increasing their therapeutic potential (106).

In brief, the study of EVs in interkingdom interactions requires careful consideration of their strain- and dose-dependent effects as well as their context-specific role. *In vitro* studies show that the elicited responses may depend on experimental conditions, such as the choice of cell lines, proinflammatory inducers and measurement methods. Explicit comparison between EVs and their parent bacteria as well as specific effector molecules enriched in EVs will hopefully reveal their mechanism of action and mechanistic targets. Key challenges include identifying biomarkers to track EV distribution and understanding the synergy of EVs with other treatments to further elucidate the complexity of their interactions.

## 5 Limitations in current research, open questions and possible future applications

Bacterial extracellular vesicles hold considerable promise yet realizing their full potential and establishing robust scientific evidence for their application requires addressing existing gaps and inconsistencies. Some of the issues discussed here, that should be considered for future work, are methods for EV purification and measurement of results, appropriate controls for adverse or non-specific effects, as well as concentration and routes of administration. We anticipate that future research will address the mechanistic effects and host interaction dynamics of EVs and their distribution in the body in more detail. Finally, we briefly discuss the transition of pEVs into future applications like their role in drug delivery or food and nutrition.

### 5.1 Study design and methodology in future pEV research

**Figure 2:**
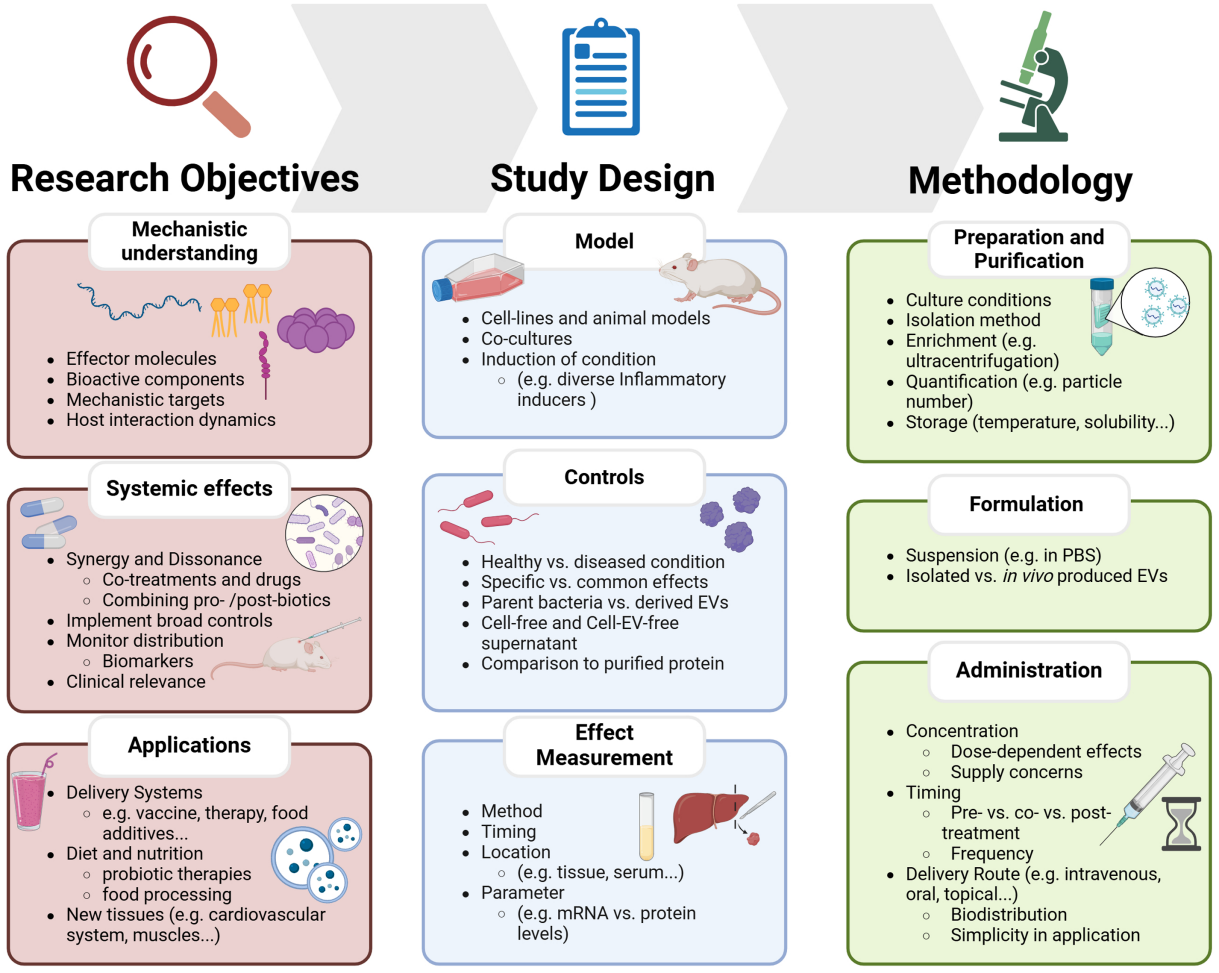
Overview of key considerations and current limitations for studying probiotic-derived extracellular vesicles. The framework is divided into three main sections: research objectives, which define the key aims such as identifying bioactive components, understanding systemic effects, or exploring EVs as delivery systems; Study Design, which outlines experimental considerations including model selection, control conditions, and effect measurement; and methodology, which covers EV preparation, formulation, and administration strategies.

EV isolation and purification methods need to be standardized to ensure reliability and allow for comparison between bacterial strains. Different isolation techniques can yield vesicles with varying composition and properties, therefore it is important to assess the chosen method’s efficiency, purity, and preservation of vesicle integrity to ensure accurate interpretation of results (107). A possible improvement is including size-exclusion based separation for removal of co-isolated protein impurities. This approach has previously significantly improved the purity and anti-inflammatory activity of EVs produced by Gram positive and Gram negative probiotic bacteria (108). Establishing common criteria and methodologies will ensure the reproducibility of findings, advancing the field collectively as summarized in Figure 2.

Some open questions and limitations revolve around the surrounding and culture conditions in which EVs are formed or processed. These variations in conditions could lead to differences in EV production and function. Monitoring specific beneficial functions of probiotics as well as their EVs is difficult due to a lack of information on their *in vivo* metabolic activities. Additionally, distinguishing effects produced by delivered probiotic EVs from those of indigenous microbes with similar effects complicates the selection of a specific strain for targeted applications (109, 110). This complexity further hampers reproducibility, given the interactions with other indigenous strains. Similarly, the properties of EVs produced by bacteria under *in vitro* conditions may differ from those produced *in vivo*, emphasizing the importance of considering intended application and administration. If administration of live bacteria is anticipated, studies should include EVs produced under conditions that closely mimic gastrointestinal settings. This consideration is crucial, especially given previous evidence that growth medium and culture conditions significantly influence EV composition and quantity (31, 32). Purification and isolation methods may not be entirely effective in separating EVs from protein aggregates or membrane fragments, therefore additional steps and measures must be implemented to ensure the quality of the end product (111–113). Failure to do so may introduce biases in the results.

Another source for bias, related to EV purification, could be the process of vesicle uptake. Different internalization routes result in the sorting of vesicle cargo to specific subcellular locations and determining the factors influencing route selection remains an unresolved question. For instance, the uptake and distribution of EVs may hinge on their size, a characteristic that could be influenced by the purification and concentration methods used, potentially leading to the enrichment of specific EV subpopulations with distinct functions (37, 113).

The importance of standardizing criteria and methods to guarantee the traceability and integrity of the results cannot be overemphasized. The heterogeneity of methods also concerns the measurement of results and parameters such as cytokine levels, which are often difficult to compare within a specific context at the time being. In this case, it is important to reason at what level—gene, protein, or three-dimensional organization—to measure, at which time-point and what location in the organism. Similar guidelines will have to be established for the induction of the condition and the start and interval of EV treatment. In both cases, the concentration administered, the duration and timing of treatment (pre- and co-treatment or post-treatment) must be carefully considered and systematically compared. Since results are often dose-dependent and the doses of EVs used vary in a range of several orders of magnitude (circa 0.1 to >1,000 μg/kg *in vivo* and 0.01 to 200 μg/mL *in vitro*), a range of dosages should be included and compared in all studies where there is no prior work available to reasonably base dose-estimations on (57, 71, 74).

In general, direct comparisons between disease-models, between bacterial strains and exploration of combination therapies as well as addressing the nuances of simultaneous secondary conditions remain limited. In addition, meaningful comparisons with drugs already on the market will provide a reference for the potential impact of EVs in clinical practice and will help to guide interpretations. For example, treatment with *Lactobacillus*-EVs and imipramine, a tricyclic antidepressant, showed similar antidepressant-like effects in mice (71). The selection of administration routes, with a preference for simple and safe methods like oral or topical administration warrants a deeper understanding of the distribution and systemic effects in the host. To ensure the safe and efficient application of EVs in clinical settings, there is an urgent need for reliable biomarkers to monitor their distribution. To date, studies have predominantly focused on understanding the functions and effects of EVs at specific sites of interest, few studies included controls for side effects.

Looking ahead, it is crucial to invest in identifying biologically active components associated with vesicles and understanding the targets on human cells they interact with. Information is available on the mechanistic properties of how selected probiotics and postbiotics interact with the human host, and it seems reasonable that these mechanisms at least partially overlap with those employed by bacterial vesicles (18, 114, 115). Although numerous reports suggest molecules and pathways involved in these EV-transferred functions, detailed information is still lacking, necessitating an effort in that direction in coming work (29). This knowledge not only aids in understanding how these vesicles contribute to maintaining homeostasis, enhancing immune function, or promoting tissue repair but also helps discern the differences between specific probiotic effects and common traits of non-pathogenic bacterial vesicles when applied strategically. In some cases, opposing effects have been observed in different contexts, emphasizing the importance of comparisons between control groups and experimental conditions. Including appropriate controls, such as vesicle-depleted supernatants or vesicles from non-beneficial bacteria helps delineate the specific effects of EVs. Lastly, understanding the experimental context, such as the microbial environment, host cell type, and physiological conditions contributes to the contextual relevance of the findings. Hence, we want to advise combining *in vitro* experiments with *in vivo* counterparts whenever possible.

### 5.2 Promising EV research targets—EV applications in delivery, diet, and food

A future avenue touched upon earlier is the use of engineered EVs as targeted delivery systems. Engineered bacterial EVs have shown promise as vaccine delivery vectors and as carriers in biomedical research (116, 117). For instance, *Lc*. *rhamnosus* GG vesicles were modified to deliver intrinsic miRNA specifically to bone tissues as treatment of osteoporosis, and *E. coli*-derived OMVs have been used as transport system for chemotherapeutic drugs (106, 118). But not only in the therapeutic space, also in the food industry could EVs be applied as a convenient delivery system. Their biocompatibility and intrinsic ability to transport metabolic components and influence intestinal barrier permeability could be exploited to increase the bioavailability of food additives with limited stability or solubility, thus contributing to improving the nutritional value of foods (119). *Lc*. *paracasei* EVs for instance were observed to aggregate or fuse with milk EVs, potentially changing the availability of associated beneficial effector molecules like the proteins p40 and p75 (120). The relationships between diet, gut microbiome and host are complicated, and both diet- and host-derived bioactive components like microRNAs could modulate the gut microbiota and its EV composition and jointly influence the availability and uptake efficiency of EVs, dietary metabolites and components (121, 122). This application, though promising, warrants an effort to know effector molecules and bioactive components present in or on the EVs and precisely understand their interaction with each other and the host.

The fact that microbial, especially probiotic EVs, interact closely with other microorganisms, food components and host systems has led to a keen interest in their use to enhance the therapeutic potential of certain foods, marking a step towards establishing functional foods and targeted probiotic therapies. As mentioned previously, pEVs can have substantial implications in the treatment of metabolic diseases like obesity and diabetes (52, 73). Since dietary habits were shown to impact amount, size, and content of gut microbiota–derived EVs, this as well concludes implications for the prevention and treatment of such metabolic diseases (110). For example, succinate production triggered by dietary protein intake, or *β*-mannan administration as an example for carbohydrate-rich food, were shown to influence the production and composition of EVs produced by bacteria (123, 124). Therefore, research should aim at understanding how these processes can alter the bioactive properties of vesicles. In this regard, comprehensive metabolomic studies could help link the presence of specific EVs to the health effects of diet.

In addition to dietary habits, the production of these foods will also affect their nutritional value and beneficial effects on health and wellbeing. EVs together with other postbiotics are naturally found in fermented foods and are used as functional components in the food industry (125). While much remains to be discovered about the role of EVs produced by microbial communities in fermented foods, these vesicles likely contribute to microbial community dynamics by modulating growth, metabolism, and resilience of the consortia that drive fermentation. Gaining insights into microbial communication within food ecosystems may ultimately enhance the development of fermented products with improved safety, nutritional quality, and health-promoting benefits for consumers (126). Effects of food processing techniques such as drying however might impact EVs’ structure, content and biodistribution requiring future mechanistic investigation and establishment of biomarkers. Beyond their given use in some fermented foods, pEVs could also be used deliberately in food production, for example as natural antibacterial agents. For example, *Lp*. *plantarum*-derived EVs have been utilized to enhance the quality of tuna fish, showcasing their efficacy in food storage technology. These vesicles did inhibit the growth of *Shewanella putrefaciens*, a strain which can cause food poisoning and food spoilage (127). Similarly, *Lc*. *casei* BL23 EVs have demonstrated inhibition of the formation of *Salmonella enteritidis* biofilms on polystyrene surfaces, hinting at potential applications in both healthcare and the food industry (128). Further, pEVs may be used as natural treatment and protection against bacterial infections during meat production, avoiding overuse of antibiotics (96). Finally, in addition to extending EV applications to non-human organisms, we would like to emphasize that it could be lucrative to extend research in humans to new tissues and organs such as the cardiovascular or respiratory system and muscles, as presented studies have shown that pEVs also accumulate at these sites after systemic administration (67, 77).

However, as in all research areas discussed here, the same main limitations considering mechanistic understanding, composition variability, host-interactions and clinical relevance must be overcome, as summarized in Figure 2. Little guidance is currently available, and long-term studies are needed to assess possible adverse effects in the human body, be it in the area of precision nutrition, viral infection or cancer therapeutics. Addressing these gaps through rigorous research and standardized methods is key to unlocking the full potential of probiotic extracellular vesicles and enabling their well-characterized, safe and effective use in clinical, biotechnological and dietary applications.

## Glossary

CRST: Chronic Restraint Stress Treatment
DSS: dextran sodium sulfate
ELISA: enzyme-linked immunosorbent assay
EPEC: enteropathogenic *E. coli*
EV: extracellular vesicle
HMECs: Human Mammary Epithelial Cell
IBD: inflammatory bowel disease
IECs: intestinal epithelial cells
IECs: intestinal epithelial cell
LAB: lactic acid bacteria
LC–MS/MS: Liquid Chromatography Tandem Mass Spectrometry
LPS: lipopolysaccharide
MALDI-TOF: Matrix-assisted laser desorption ionization–time of flight mass spectrometry
MAMPs: microbial-associated molecular patterns
NOD: nucleotide-binding oligomerization domain
OMVs: outer membrane vesicles
PAMPs: pathogen-associated molecular patterns
pEV: probiotic-derived EV
PRRs: pattern recognition receptors
ROS: reactive oxygen species
RT-qPCR: quantitative reverse transcription polymerase chain reaction
T1D: Type 1 diabetes
Th: T helper cell
TJ: tight junction
TLR: Toll-like receptor
Treg: regulatory T cell
